# Direct Comparison of the Safety and Efficacy of Ferric Carboxymaltose versus Iron Dextran in Patients with Iron Deficiency Anemia

**DOI:** 10.1155/2013/169107

**Published:** 2013-08-29

**Authors:** Iftikhar Hussain, Jessica Bhoyroo, Angelia Butcher, Todd A. Koch, Andy He, David B. Bregman

**Affiliations:** ^1^Vital Prospects Clinical Research Institute, PC, Tulsa, OK 74136, USA; ^2^Luitpold Pharmaceuticals, Inc., Norristown, PA 19403, USA; ^3^Department of Pathology, Albert Einstein College of Medicine, Bronx, NY 10461, USA

## Abstract

Several intravenous iron complexes are available for the treatment of iron deficiency anemia (IDA). Iron dextran (DEX) is associated with an elevated risk of potentially serious anaphylactic reactions, whereas others must be administered in several small infusions to avoid labile iron reactions. Ferric carboxymaltose (FCM) is a nondextran intravenous iron which can be administered in high single doses. A randomized, open label, and multicenter comparison of FCM to DEX in adults with IDA and baseline hemoglobin of ≤11.0 g/dL was conducted. A total of 160 patients were in the safety population (FCM *n* = 82; DEX *n* = 78). Adverse events, including immune system disorders (0% in FCM versus 10.3% in DEX, *P* = 0.003) and skin disorders (7.3% in FCM versus 24.4% in DEX, *P* = 0.004), were less frequently observed in the FCM group. A greater portion of patients in the FCM group experienced a transient, asymptomatic decrease in phosphate compared to patients in the DEX group (8.5% in FCM versus 0% in DEX, *P* = 0.014). In the FCM arm, the change in hemoglobin from baseline to the highest observed level was 2.8 g/dL, whereas the DEX arm displayed a change of 2.4 g/dL (*P* = 0.20). Treatment of IDA with FCM resulted in fewer hypersensitivity-related reactions than DEX.

## 1. Introduction

Iron deficiency is one of the most common deficiencies in the world and can lead to anemia [1–3]. Patients with reduced absorption of dietary iron (e.g., patients with inflammatory bowel disease, gastrointestinal surgery, or who have had gastric bypass) or patients with increased utilization or loss of iron from the body (e.g., pregnancy/childbirth, heavy uterine bleeding, lactation, hemodialysis, or surgery) are particularly at risk [1, 4–6]. IDA may adversely affect cognitive function, physical activity, immune response, inflammatory conditions, and pregnancy outcomes [7]. Different kinds of therapies from oral iron to blood transfusion are currently used to treat IDA. The aim of the treatment is to return both hemoglobin and iron stores to normal levels. Oral iron therapy is the treatment of choice for the majority of patients with IDA because of its effectiveness, safety, and low cost. Even though many patients can tolerate oral iron without difficulty, up to 40% may have side effects attributable to oral iron replacement therapy [8]. The incidence of side effects increase with the dose and adversely affects compliance. Also, oral iron is often not capable of replenishing severe iron deficits [9]. As a treatment of last resort, blood transfusions are used, but they may lead to the transmission of known and unknown pathogens, immunological impact, and transfusion reactions. 

Intravenous (IV) administration of iron is preferred in patients who are unable to absorb sufficient iron from the gastrointestinal tract or those who do not tolerate oral iron, in patients in whom blood transfusions need to be avoided for medical or religious reasons, and in patients with chronic iron loss exceeding the rate of replacement possible with oral treatment. First generation intravenous iron formulations, namely, iron dextran (DEX), have been used as an alternative to oral iron in the treatment of IDA of multiple etiologies although it is known to cause severe immunological responses, including fatal anaphylactic reactions [3, 4, 10]. Second generation, nondextran IV irons, such as iron sucrose and sodium ferric gluconate, do not contain dextran, or modified dextran, but they have significant dosage and administration rate limitations. They are characterized by a risk of adverse reactions called labile iron reactions at higher doses which may include hypotension, cramping, diarrhea, or chest pain [11]. Feraheme (ferumoxytol) is a recently approved IV iron administered as two injections of 510 mg [2]. Although Feraheme was designed with a modified dextran shell to reduce immunogenic potential, anaphylaxis in individuals with previous hypersensitivity to iron dextran has been reported [12]. Ferric carboxymaltose (FCM), a novel IV iron, is a stable Type I polynuclear iron (III) hydroxide carbohydrate complex [1] that has been approved in Europe for the treatment of iron deficiency since 2007. After IV administration, FCM is mainly found in the reticuloendothelial system of the liver, the spleen, and bone marrow [1, 13]. The iron slowly dissociates from the complex and can be efficiently used in the bone marrow for hemoglobin synthesis. FCM offers significant advantages compared to earlier generation IV iron preparations. Due to its structure, FCM is more stable than sodium ferric gluconate and iron sucrose. It is therefore possible to administer much higher single doses over shorter periods of time than sodium ferric gluconate or iron sucrose, resulting in the need for fewer administrations to replete iron stores [1, 4, 5, 13–15]. In addition, it is not a dextran or modified dextran so the risk of hypersensitivity reactions is reduced. In multiple studies, FCM has been shown to be an effective option in the treatment of IDA, and it also improves the quality of life of patients [1, 4, 5].

The aim of this study was to conduct a head-to-head comparison of the safety of an investigational IV iron regimen (FCM) versus IV iron dextran in the treatment of IDA when oral iron was not tolerated or the response was poor. 

## 2. Materials and Methods

**Figure 1:**
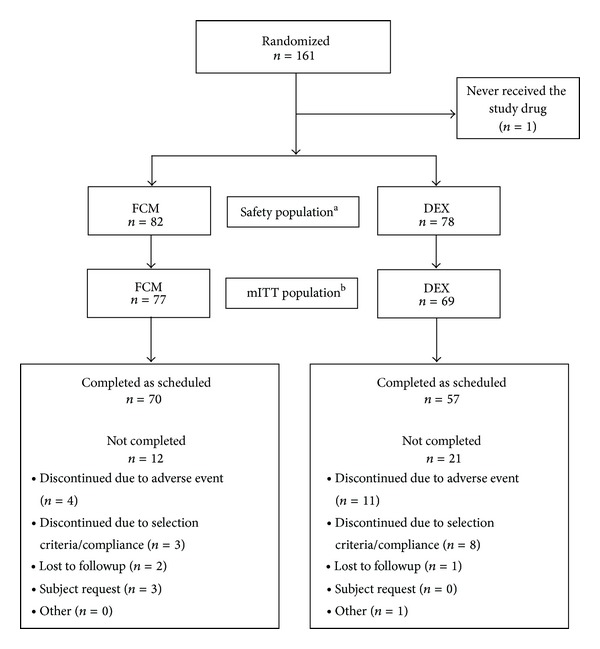
Flowchart of the study. ^a^All subjects treated with FCM or DEX were included in the safety population. ^b^mITT: all subjects in the safety population with 2 baseline hemoglobin values (with <1 g/dL difference between the 2 values) and at least 1 postbaseline hemoglobin value on or before Day 49 or intervention date, whichever came first.

This was a Phase 3b, 7-week, multicenter, open label, randomized (1 : 1) study that evaluated the safety and efficacy of FCM versus DEX. It took place between July 2008 and July 2009 and involved 27 sites within the United States. The protocol was approved by the Institutional Review Board for each site, and the trial was performed in accordance with the Declaration of Helsinki. Informed content was acquired from each patient prior to inclusion of the study. A flowchart of the study is depicted in Figure 1. Clinical trials registration information: ClinicalTrials.gov number, NCT00704028.

The study treatment involved a single maximum dose (15 mg/kg body weight up to 750 mg) administered weekly until the total iron requirement (calculated by the Ganzoni formula) [16] or a maximum of 2,250 mg was reached. Patients were 18 years old and older with IDA and had a history of intolerance to oral iron or an unsatisfactory response to oral iron. The screening visit hemoglobin had to be ≤11.0 g/dL, and the screening ferritin level had to be ≤100 ng/mL or ≤300 ng/mL when transferrin saturation (TSAT) was ≤30%. 

 Patients were excluded if they had a history of a hypersensitivity reaction to any component of FCM or DEX, required dialysis for treatment of CKD, had anemia not due to iron deficiency, were previously treated with IV iron, had red blood cell transfusion(s) or antibiotics during the 10 days prior to screening, were treated with erythropoiesis stimulating agents in a regimen exceeding product labeled dosing during the 30 days prior to screening (or during the study), were receiving treatment with radiotherapy and/or chemotherapy, or required a surgical procedure that necessitated general anesthesia. Additional exclusion criteria included infection other than viral upper respiratory tract infection, aspartate aminotransferase (AST) or alanine aminotransferase (ALT) >1.5 times the upper limit of normal, active hepatitis, human immunodeficiency virus positive, being treated for asthma, recent alcohol abuse, history of hemochromatosis or other iron storage disorders, systolic blood pressure ≥180 or <80 mmHg or diastolic blood pressure ≥100 or <40 mmHg at screening or Day 0, significant cardiovascular disease, breastfeeding, and sexually active females not willing to use an acceptable form of contraception.

After confirmation of eligibility, study site personnel randomly allocated participants using an integrated voice response system. Treatment assignments to study drugs were randomly generated in blocks of 4. Patients were stratified by baseline hemoglobin (≤8, 8.1–9.5, ≥9.6), baseline cardiovascular risk (Category 1–4), and use of immunosuppressive therapy. Cardiovascular risk was defined by using variables from the Framingham model [17]. Risk score was defined as Category 1 if there was no known risk factor, Category 2 if the subject had one of the following: age > 75, current smoker, hypertension or on antihypertensive medications, hyperlipidemia or use of a lipid lowering medicine, use of low dose aspirin, Category 3 if they had one of the following: diabetes or ≥2 of the risk factors used to define Category 2, and Category 4 if they had prior history of cardiovascular disease.

Patients who were randomized to FCM (Luitpold Pharmaceuticals, Inc.) received their study drug on Days 0, 7, and 14. Both of the doses of these two study drugs were calculated by the Ganzoni formula. The IV iron dose was calculated by the following algorithm: weight in kg × (15-current hemoglobin g/dL) × 2.4 + 500 = total iron requirement in milligrams (mg). The maximum total was not to exceed 2,250 mg. If the subject was postpartum, the prepregnancy weight was used. If TSAT >20% and ferritin >50 ng/mL, 500 mg was subtracted. Patients received up to 750 mg of iron as undiluted FCM (15 mg/kg up to a maximum of 750 mg) at 100 mg per minute via IV push injection weekly until the calculated iron deficit dose had been administered.

Patients who were randomized to DEX received their study drug between Days 0 and 42. On Day 0, a test dose of 25 mg of DEX was administered slowly over 5 minutes, and the subject was observed for 15 minutes to 1 hour, and if no reaction occurred, the remainder of the Day 0 dose was given. The total DEX dose was determined by calculating the total iron requirement and dividing this total amount into one or more single dose(s). They received DEX at doses and infusion times as determined by the investigator until the calculated iron deficit dose was administered (to a maximum cumulative dose of 2,250 mg). Investigators were allowed to administer either Dexferrum (American Regent, Inc.) or INFeD (Watson Pharma, Inc.). The dose and rate of administration of the DEX was up to the discretion of the investigator as this would mimic practice in the clinical setting.

The primary endpoint was the incidence of treatment-emergent serious adverse events (AEs) from Day 0 to Day 42, or 28 days after the last dose of study drug. Secondary endpoints included incidence of treatment-emergent AEs, incidence of treatment-emergent abnormal clinical laboratory values, and incidence of potentially clinically significant vital sign values. Assessments for safety were performed on Days 0, 7, 14, 28, and 42. Measurements of safety included AEs, physical examination, vital signs, and clinical laboratory evaluations. Adverse events were collected from the time of the initial treatment of FCM or DEX on Day 0 to the end of the study (Day 42), or 28 days after the last dose of study drug, whichever was longer. Electrocardiogram (ECG) assessments were performed at screening, Days 0 and 42. A follow-up phone call to collect AEs may have been required for patients that discontinued early.

Adverse events were classified using the Medical Dictionary for Regulatory Activities Terminology. For the purpose of this study, allergic reactions (hypersensitivity) were classified by grade according to the National Cancer Institute (NCI) Common Terminology Criteria for Adverse Events (CTCAE), Version 3.0 [18]. The investigator classified the severity of all AEs as Grades 1 to 5 by CTCAE criteria if they existed or as Grade 1 if considered mild, Grade 2 if moderate, Grade 3 if severe, Grade 4 if life-threatening, and Grade 5 if the AE resulted in death. An AE was classified as serious if it met any one of the following: death, life-threatening, hospitalization, disability, congenital anomaly/birth defect, or important medical events. In addition, the investigator documented his/her opinion of the relationship of the event to the study drug either as none, unlikely, possible, or probably. Adverse events that followed the treatment with study drug were classified as treatment-emergent AE.

In order to assess the development of clinically significant signs and symptoms, the patients were evaluated prior to drug administration. Sitting heart rate and blood pressure were assessed before, immediately after, and 30 minutes after administration. Patients were monitored for serious acute reactions as hypersensitivity or labile iron reactions to IV iron products which have rarely been reported.

Efficacy measures included change in hemoglobin, ferritin, and TSAT from baseline to the highest value observed for all patients in the Modified Intent-to-Treat (mITT) population. Hematology and iron indices were measured at screening, baseline, Days 0, 7, 14, 28, and 42.

### 2.1. Statistical  Analysis

No formal sample size calculation was performed for this trial, the primary objective of which was to assess the safety of FCM. However, the statistical precision of the endpoints (as measured by confidence interval width) was adequate with the planned sample size. Approximately 100 patients (up to 200) were expected to be randomized. Enrollment was ceased when 160 patients were randomized because subsequent to discussions with the FDA, two other efficacy and safety trials were initiated that fulfilled the same programmatic goals as this trial (ClinicalTrials.gov nos. NCT00981045 and NCT00982007). These trials did not compare FCM to iron dextran. 

All patients were included in the safety population, which was defined as patients who received a dose of randomized FCM or DEX. All safety analyses were performed using the safety population. The mITT population was defined as patients from the safety population who had two baseline hemoglobin values and at least 1 postbaseline hemoglobin value on or before the Day 42 visit (no later than Day 49) or date of first intervention, whichever was earlier.

All statistical tests were two tailed and performed with a 0.05 Type I error. Data comparisons were performed using the one-way analysis of variance (ANOVA) and Fisher's exact test. The use of mean standard deviations (SD) was preferred as the use of the standard error mean. Some results were reported in percentages, and some data are mean +/− (SD). *P* values < 0.05 were considered statistically significant.

## 3. Results

A total of 160 patients were randomized to the FCM or DEX group. No statistically significant differences were observed between the FCM and DEX groups in the safety population for any of the demographic characteristics (Table 1). Most of the patients were females with a primary etiology of heavy uterine bleeding, inflammatory bowel disease, or other gastrointestinal pathologies. The majority of patients had a cardiovascular risk of 1 or 2 and had a poor response to iron therapy. Similar baseline values of mean hemoglobin and TSAT were observed between both FCM and DEX groups. A higher mean ferritin value at baseline was observed for patients in the DEX group compared to the FCM group; however, this difference did not reach statistical significance (*P* = 0.067) and was not of a magnitude considered to be clinically significant. In the safety population for subjects receiving FCM, the mean (±SD) total dose received during the study was 1450.9 (±372.02) mg and 1342.5 (±603.06) mg for patients receiving DEX. Of the patients that received FCM, 58.5% received 1-2 IV push injections and patients that received DEX, 57.7% received 1-2 infusions. In the DEX group, 32.1% of the patients received one infusion. Of the patients who were randomized to receive DEX, 73 patients received Dexferrum and 5 patients received INFeD. The mean single dose in the FCM group was 628.6 mg and in the DEX group was 649.5 mg.

The most commonly (≥10.0%) experienced treatment-emergent AEs in the DEX group were headache and nausea; the only treatment-emergent AE experienced by ≥10.0% of patients in the FCM group was nausea (Table 2). The majority of the treatment-emergent AEs experienced during the study were classified by the investigators as severity Grades 1 or 2. Compared to the FCM group, a statistically significantly greater proportion (*P* < 0.01) of patients in the DEX group experienced AEs associated with immune system disorders (i.e., hypersensitivity) or skin and subcutaneous tissue disorders (i.e., urticaria). Adverse events related to the immune system were classified by the investigators as severity of Grades 2, 3, or 4 and AEs related to skin and subcutaneous tissue disorders classified as severity of Grades 1 or 2. 

 In regard of serious AEs, 5 patients (6.1%) in the FCM group and 3 patients (3.8%) in the DEX group experienced at least 1 serious adverse event. Of the 5 subjects that experienced a serious AE in the FCM group, the causality was none in 4 subjects and unlikely in one subject. On the other hand, the causality of serious AEs in the DEX group were probable in 2 patients and none in 1 patient. A summary of subjects who experienced serious AEs is listed in Table 3.

One death occurred in the FCM group during the study which the investigator considered to be unrelated to study drug. The patient was a 76-year-old man with a history of coronary artery disease, myocardial infarction, hypertension, and Crohn's disease with small bowel resection who received a single dose of 750 mg of FCM. The cause of death, which occurred 28 days after the dose of FCM, was stated to be “possible myocardial infarction.”

The majority of the AEs in patients who were prematurely discontinued were considered possibly or probably related to study drug. Six patients (7.3%) in the FCM group and 11 patients (14.1%) in the DEX group were discontinued from the study drug due to the occurrence of drug related AEs. Four patients (4.9%) in the FCM group and 11 (14.1%) patients in the DEX group were discontinued from the study due to occurrence of AEs (Table 4). The majority of the AEs leading to discontinuation from study for patients in the DEX group were due to hypersensitivity reactions. For the FCM group, causality ranged from none to probable, and the AEs varied. 

**Figure 2:**
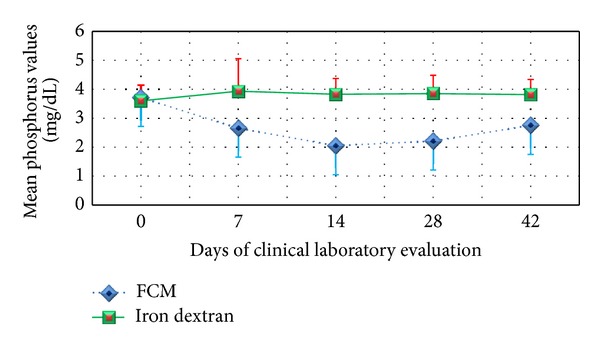
Mean phosphorus values for FCM and DEX patients.

One of the notable differences in clinical chemistry values reported between the groups was for the proportion of patients with significant phosphorus values below 2.0 mg/dL. Compared to the DEX group, a statistically significantly greater proportion of subjects in the FCM group experienced a transient decrease in serum phosphate (8.5% versus 0%, *P* < 0.05). A statistically significantly greater mean decrease from baseline to final value (*P* ≤ 0.001) was observed in the FCM group (Table 5).  Figure 2 illustrates the phosphorus change from Day 0 to Day 42. The mean value reached its nadir (2.05 mg/dL) at Day 14 and was within the normal range (2.5–4.5 mg/dL) by Day 42.

Statistically significantly greater mean increases from baseline to final value were observed in the FCM group compared to the DEX group for alkaline phosphatase (*P* = 0.023). Mean ALT and AST increased form baseline to final value (*P* = 0.052 and *P* = 0.051, resp.) in the FCM group but remained within their respective normal ranges. Thus, these increases were not considered clinically significant (Table 5).

**Figure 3:**
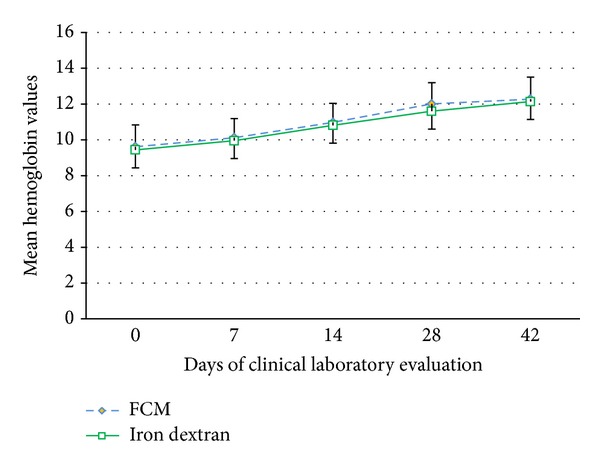
Mean hemoglobin values for FCM and DEX patients.

Regarding measures of efficacy, there were no statistically significant differences in mean hemoglobin increase between FCM and DEX. The change in hemoglobin from baseline to the highest value was 2.8 g/dL in the FCM arm and 2.4 g/dL in the DEX arm (*P* = 0.200). Within each group, there were statistically significant increases in hemoglobin for both FCM and DEX from baseline to the highest value observed (*P* = 0.001). Change in hemoglobin is displayed in Figure 3. Mean changes in ferritin from baseline to the highest value observed was higher in the FCM group than in the DEX group (543.2 ng/mL versus 319.7, *P* = 0.001, resp.). In regards to mean change in TSAT, a greater difference from baseline to highest value was observed in the DEX group than the FCM group (38.1% versus 29.6%, *P* = 0.012, resp.). A detailed summary is shown in Table 6.

## 4. Discussion

The primary objective of this trial was to compare the safety of FCM to DEX in patients with IDA. Most of the patients were females whose principal cause of anemia was heavy uterine bleeding, inflammatory bowel disease, or other gastrointestinal pathologies. This trial confirmed the safety profile of FCM in comparison to another frequently administered IV iron, iron dextran. 

Although the trial had a sample size of only 160 patients, several adverse events related to immune system disorders and skin disorders were observed more frequently in the DEX group than the FCM group. Two subjects in the DEX group reported serious AEs that were considered to be study drug related as opposed to none in the FCM group. Moreover, about twice as many subjects in the DEX group were prematurely discontinued from study drug due to the occurrence of an AE (11 versus 4). The majority of AEs resulting in premature discontinuation from study drug were considered possibly or probably related to study drug. Many in the DEX group were related to allergy/immune response.

Many studies have shown that FCM has a relatively modest incidence of adverse effects [4, 4, 19]. The most commonly observed AEs were nausea, headache, abdominal pain, diarrhea, and rash [1, 13]. Serious AEs observed were considered to be unrelated to the administration of FCM [5, 13]. Iron dextran is currently the only IV iron approved in the US for use in ID patients (i.e., other than chronic kidney disease). It can lead to potentially dangerous clinical scenarios because of the possibility of inducing a severe immunological response. The risk of anaphylaxis/anaphylactoid reactions caused by DEX has been reported previously [10, 20]. The iron dextran utilized in this trial was mainly high molecular weight iron dextran (Dexferrum). The difference between the high molecular weight and low molecular weight iron dextran formulations is due to the size of the iron cores, not the dextran shell, the suspected component responsible for iron dextran-related anaphylactic reactions [21]. The amounts of carbohydrate shell are approximately equal in the two iron dextrans [22, 23]. Studies have found that high molecular weight iron dextran compared with low molecular weight iron dextran had higher frequencies of anaphylactic-type reactions [24, 25], while another study found similar or higher frequencies with low molecular weight iron dextran [26]. The current study indicates a favorable safety profile for FCM as compared to that of high molecular weight iron dextran due to the small number of subjects that received low molecular weight iron dextran. 

Compared to the DEX group, the proportion of patients experiencing a transient decrease in serum phosphate was higher in the FCM group. The mean value of phosphorus decreased from Day 0 to Day 14 in patients in the FCM group but increased after Day 14 to Day 42. The mean value at Day 42 (2.75 mg/dL) is within the normal range for the central laboratory used in this trial (2.2–5.1 mg/dL). None of the patients with reduced phosphorus levels were clinically symptomatic or received any medications to treat these changes in phosphorus level. A transient decrease in serum phosphate has also been reported in other trials of FCM [4, 5, 27, 28]. A possible mechanism is that FCM transiently increases the levels of intact fibroblast growth factor 23, which is a hormone that inhibits renal reabsorption of phosphate [29].

FCM was equivalently effective as DEX in the treatment of IDA. Both treatments improved hemoglobin by an average of approximately 2.5 g/dL over the study period, returning hemoglobin from starting value of about 9.5 g/dL to about 12 g/dL in around 4 weeks after first dose. Intravenous administration of FCM with doses of up to 750 mg delivered at a rate of 100 mg per minute also significantly increased ferritin level. As compared to iron dextran, FCM had a greater effect with respect to the restoration of iron stores (as indicated by an increase in ferritin).

 Our results, consistent with the results of previous clinical trials, [1, 3, 4, 13, 30, 31] demonstrate that FCM is safe and well tolerated. In the current study, consistent with previous trials [4, 5, 13, 15, 30–32], FCM increased hemoglobin levels, replenished iron stores, and had a low incidence of AEs. In this study there was also a lower rate of allergic reactions in the FCM group with respect to the DEX group. Moreover, large doses of FCM (up to 750 mg) could be administered via rapid IV push injection (at 100 mg per minute) in this trial. Thus, FCM is a safe, effective, and convenient option for the treatment of IDA of any etiology [1, 5, 32].

## Figures and Tables

**Table 1:** Baseline demographics and characteristics.

Demographic characteristic	FCM (*N* = 82)	DEX (*N* = 78)	*P* value
Age (years)			
Mean (SD)	46.2 (14.64)	48.2 (17.10)	*P* = 0.422
Minimum, maximum	20, 86	18, 84	
≤65	72 (87.8%)	63 (80.8%)	
66–75	5 (6.1%)	9 (11.5%)	
76–85	5 (6.1%)	6 (7.7%)	
Gender			
Female	73 (89.0%)	69 (88.5%)	*P* = 1.00
Male	9 (11.0%)	9 (11.5%)	
Race			
African American	27 (32.9%)	21 (26.9%)	*P* = 0.639
Asian	4 (4.9%)	3 (3.8%)	
Caucasian	41 (50.0%)	42 (53.8%)	
Hispanic	7 (8.5%)	11 (14.1%)	
Other	3 (3.7%)	1 (1.3%)	
Iron intolerance			
No	37 (45.1%)	33 (42.3%)	*P* = 0.752
Yes	45 (54.9%)	45 (57.7%)	
Weight (kg)			
Mean (SD)	79.89 (22.960)	82.18 (20.681)	*P* = 0.509
Height (cm)			
Mean (SD)	163.65 (7.965)	163.80 (8.245)	*P* = 0.903
Drug allergy			
No	44 (53.7%)	45 (57.7%)	*P* = 0.636
Yes	38 (46.3%)	33 (42.3%)	
Baseline hemoglobin (g/dL)			
Mean (SD)	9.63 (1.190)	9.49 (1.260)	*P* = 0.472
Hemoglobin category			
≤8.0 g/dL	9 (11.0%)	9 (11.5%)	*P* = 0.857
8.1–9.5 g/dL	23 (28.0%)	20 (25.6%)	
≥9.6 g/dL	50 (61.0%)	49 (62.8%)	
Use of immunosuppressive therapy			
No	79 (96.3%)	77 (98.7%)	*P* = 0.621
Yes	3 (3.7%)	1 (1.3%)	
Cardiovascular risk			
1	41 (50.0%)	40 (51.3%)	*P* = 0.809
2	19 (23.2%)	19 (24.4%)	
3	12 (14.6%)	10 (12.8%)	
4	10 (12.2%)	9 (11.5%)	
Baseline TSAT (%)			
Mean (SD)	10.05 (10.010)	10.95 (11.257)	*P* = 0.596
<20	72 (87.8%)	63 (80.8%)	
Baseline ferritin (ng/mL)			
Mean (SD)	12.89 (21.848)	23.12 (44.828)	*P* = 0.067
<100	80 (97.6%)	71 (91.0%)	
Past response to iron therapy			
Poor/no	17 (20.7%)	18 (23.1%)	*P* = 0.849
Poor/yes	65 (79.3%)	60 (76.9%)	
Etiology of IDA			
Heavy uterine bleeding	32 (39.0%)	27 (34.6%)	*P* = 0.659
Chronic kidney disease	4 (4.9%)	2 (2.6%)	
IBD/Gastrointestinal related	31 (37.8%)	30 (38.5%)	
Other	4 (4.9%)	9 (11.5%)	
Postpartum	2 (2.4%)	1 (1.3%)	
Unknown	9 (11.0%)	9 (11.5%)	

IBD: inflammatory bowel disease; IDA: iron deficiency anemia; SD: standard deviation.

**Table 2:** Treatment-emergent adverse events experienced by ≥5% of patients in either FCM or DEX group.

MedDRA SOC^a^ Preferred term	FCM (*N* = 82) *n* (%)	DEX (*N* = 78) *n* (%)	*P* value^b^
At Least 1 treatment-emergent adverse event	60 (73.2%)	59 (75.6%)	0.856
Gastrointestinal disorders	24 (29.3%)	14 (17.9%)	0.099
Diarrhea	5 (6.1%)	3 (3.8%)	0.720
Nausea	12 (14.6%)	8 (10.3%)	0.477
Vomiting	5 (6.1%)	4 (5.1%)	1.000
Immune system disorders	0	8 (10.3%)	0.003^†^
Hypersensitivity	0	7 (9.0%)	0.006^†^
Metabolism and nutrition disorders	8 (9.8%)	4 (5.1%)	0.371
Hypophosphatemia	7 (8.5%)	0	0.014*
Nervous system disorders	16 (19.5%)	17 (21.8%)	0.845
Dizziness	6 (7.3%)	4 (5.1%)	0.747
Headache	6 (7.3%)	10 (12.8%)	0.297
Skin and subcutaneous tissue disorders	6 (7.3%)	19 (24.4%)	0.004^†^
Pruritus	2 (2.4%)	6 (7.7%)	0.160
Rash	2 (2.4%)	5 (6.4%)	0.268
Urticaria	0	7 (9.0%)	0.006^†^

MedDRA: medical dictionary for regulatory activities; SOC: system organ class.

^a^Each subject is counted only once per SOC when multiple preferred terms are reported for the SOC.

^b^From Fisher's exact test.

*Statistically significant at the *P* = 0.05 level.

^†^Statistically significant at the *P* = 0.01 level.

**Table 3:** Subjects who experienced serious adverse events.

Subject	Age/sex	Event	Severity	Causality
FCM				
1	63/M	Syncope	Grade 4	None
		Asthenia	Grade 4	None
2	54/F	Abdominal pain	Grade 2	None
3	76/M	Crohn's disease	Grade 3	Unlikely
		Death	Grade 5	Unlikely
4	83/F	Syncope	Grade 3	None
5	53/M	Colon cancer recurrent	Grade 4	None

DEX				
1	32/F	Hypersensitivity	Grade 4	Probable
		Atrial fibrillation	Grade 2	Probable
2	56/F	Vascular pseudoaneurysm	Grade 3	None
3	64/F	Anaphylactic reaction	Grade 4	Probable

**Table 4:** Patients who experienced adverse events that led to premature discontinuation from the study.

Subject	Age/sex	Event	Severity	Causality
FCM				
1	28/F	Injection site reaction	Grade 2	Probable
2	56/M	Systemic inflammatory response syndrome	Grade 3	Probable
3	76/M	Death	Grade 5	Unlikely
4	53/M	Colon cancer recurrent	Grade 4	None

DEX				
1	81/M	Dyspnea	Grade 4	Probable
2	19/F	Abdominal pain	Grade 1	Probable
3	29/F	Hypersensitivity	Grade 2	Probable
4	21/F	Hypersensitivity	Grade 2	Probable
5	57/F	Hypersensitivity	Grade 2	Probable
6	45/M	Hypersensitivity	Grade 3	Probable
7	37/F	Peripheral edema	Grade 3	Probable
8	32/F	Hypersensitivity	Grade 4	Probable
9	18/F	Dyspnea	Grade 1	Probable
10	64/F	Anaphylactic reaction	Grade 4	Probable
11	20/F	Hypersensitivity	Grade 3	Probable

**Table 5:** Changes in laboratory values.

Chemistry parameter (units)	FCM			DEX			*P* value^a^
	*N*	Baseline mean (SD)	Change to final value (SD)	*N*	Baseline mean (SD)	Change to final value (SD)	
ALT (SGPT) (U/L)	82	16.0 (7.09)	5.6 (9.80)	76	17.2 (12.72)	2.1 (12.77)	0.052
AST (SGOT) (U/L)	82	20.0 (7.94)	3.1 (11.41)	76	21.0 (10.04)	−0.4 (10.93)	0.051
Albumin (g/dL)	82	3.83 (0.499)	0.14 (0.313)	76	3.77 (0.446)	0.13 (0.373)	0.930
Alkaline phosphatase (U/L)	82	76.2 (36.29)	11.3 (24.40)	76	81.7 (34.46)	3.6 (16.88)	0.023
C-reactive protein (mg/dL)	82	0.7356 (1.38596)	−0.1090 (1.19793)	76	0.6104 (1.02749)	0.1315 (0.52244)	0.109
Calcium (mg/dL)	82	9.44 (0.543)	0.06 (0.412)	76	9.49 (0.469)	0.15 (0.542)	0.233
Creatinine (mg/L)	82	0.88 (0.738)	−0.04 (0.324)	76	0.80 (0.203)	−0.02 (0.095)	0.531
GGT (U/L)	82	23.1 (24.21)	8.3 (25.50)	76	24.4 (26.24)	6.5 (13.20)	0.573
LDH (U/L)	82	168.0 (39.75)	−1.9 (29.17)	76	171.4 (34.45)	−3.8 (25.35)	0.661
Magnesium (mg/dL)	82	2.06 (0.198)	0.00 (0.187)	76	2.08 (0.218)	−0.04 (0.180)	0.227
Phosphorus (mg/dL)	82	3.71 (0.712)	−0.78 (0.770)	76	3.62 (0.502)	0.22 (0.580)	≤0.001
Bicarbonate (mEq/L)	82	21.37 (3.073)	−0.19 (4.064)	76	21.35 (3.282)	−0.02 (2.845)	0.770
Chloride (mEq/L)	82	104.8 (4.31)	0.2 (3.63)	76	105.4 (3.57)	−0.4 (3.35)	0.281
Glucose (mg/dL)	82	115.7 (60.83)	−6.7 (43.09)	76	111.4 (55.87)	−6.6 (35.87)	0.976
Potassium (mEq/L)	82	4.20 (0.473)	−0.03 (0.412)	76	4.27 (0.408)	−0.05 (0.495)	0.789
Sodium (mEq/L)	82	141.5 (3.71)	−0.1 (3.85)	76	141.3 (3.34)	0.8 (3.68)	0.141
Total bilirubin (mg/dL)	82	0.33 (0.143)	0.03 (0.142)	76	0.34 (0.222)	0.01 (0.125)	0.486
Urea nitrogen (mg/dL)	82	16.5 (20.88)	−0.8 (12.48)	76	14.6 (6.03)	0.1 (4.20)	0.546

^a^ *P* value for difference between change for FCM versus DEX using a 1-way ANOVA.

**Table 6:** Changes in hemoglobin, ferritin, and TSAT values.

	FCM (*N* = 77)	DEX (*N* = 69)
Hemoglobin (g/dL)		
Baseline		
Mean (SD)	9.6 (1.18)	9.4 (1.31)
Median	9.9	9.8
Highest value		
Mean (SD)	12.4 (1.26)	11.9 (1.47)
Median	12.5	11.8
Change to highest value		
Mean (SD)	2.8 (1.44)	2.4 (1.71)
Median	2.7	2.7
*P* value (within group)^a^	0.001^†^	0.001
*P* value (versus FCM)^b^		0.200
Ferritin (ng/mL)		
Baseline		
Mean (SD)	11.6 (19.29)	21.2 (44.62)
Median	6.2	5.9
Highest value		
Mean (SD)	554.7 (287.84)	340.8 (245.46)
Median	503.5	278.8
Change to highest value		
Mean (SD)	543.2 (280.06)	319.7 (239.96)
Median	491.9	268.9
*P* value (within group)^a^	0.001^†^	0.001^†^
*P* value (versus FCM)^b^		0.001^†^
TSAT (%)		
Baseline		
Mean (SD)	10.2 (10.27)	9.6 (8.89)
Median	7.0	5.5
Highest value		
Mean (SD)	39.8 (15.45)	47.7 (20.73)
Median	39.0	48.0
Change to highest value		
Mean (SD)	29.6 (18.02)	38.1 (22.06)
Median	28.0	39.5
*P* value (within group)^a^	0.001^†^	0.001^†^
*P* value (versus FCM)^b^		0.012*

SD: standard deviation.

^a^ *P* value from the paired *t*-test for the within group change from baseline.

^b^ *P* value from 1-way ANOVA.

*Statistically significant at *P* = 0.05.

^†^Statistically significant at *P* = 0.001.
